# Relationship Between Early and Maximal Isometric Upper-Body Push and Pull Force Production Among Elite Female and Male Swedish Track and Field Throwers

**DOI:** 10.3390/sports13070226

**Published:** 2025-07-10

**Authors:** Jesper Augustsson, Ted Gunhamn, Håkan Andersson

**Affiliations:** 1 Department of Sport Science, Faculty of Social Sciences, Linnaeus University, 391 82 Kalmar, Sweden; ted.olssongunhamn@lnu.se; 2 High Performance Center, Strength and Conditioning Institute, 352 46 Vaxjo, Sweden; hakan.andersson@hpcsweden.com

**Keywords:** explosive strength, force–time characteristics, isometric performance testing, strength diagnostics, injury prevention, athlete health, biomechanics

## Abstract

Maximal and explosive strength—defined as the ability to rapidly generate high levels of force—are widely recognized as critical for performance in strength–power sports such as track and field throwing. However, their interrelationship remains insufficiently examined, particularly in the upper body of elite athletes. This study examined the relationship between early force production (≤250 ms, subdivided into early phase: 0–100 ms; late phase: 100–250 ms) and peak isometric upper-body push and pull force in elite Swedish track and field throwers. A total of 30 athletes (17 females, 13 males; aged 18–34 years), all competing nationally or internationally in discus, hammer, shot put, or javelin, participated in a cross-sectional assessment. Isometric force was measured during bench press (push) and supine bench row (pull) using a custom-built device. Force output was recorded at 50, 100, 150, 200, and 250 ms, along with peak force. The results showed a progressive increase in the correlation between force at early time points and peak force. Associations were weak to moderate at 50–100 ms (*r* = 0.07–0.55) and became strong to very strong at 150–250 ms (*r* = 0.64–0.92). These patterns were consistent across sexes and test types. The findings suggest that maximal strength becomes increasingly important as force production time extends beyond 100 ms. Coaches may benefit from assessing both early and peak force characteristics to inform strength profiling and guide training focus, though further research is needed to determine their impact on performance.

## 1. Introduction

Strength and power are widely recognized as key components of successful athletic performance [1,2], making their development a priority for many athletes and coaches [3]. High levels of maximal strength have consistently been shown to support athletic performance [4,5], and increases in force production through strength training have been noted to enhance athletic performance [6,7]. Although maximal strength underpins many performance capacities, the ability to generate force rapidly is often more critical in athletic contexts where the time available for force application is limited [8,9,10].

Athletes’ time-limited force-producing abilities—commonly referred to as explosive strength—can be assessed using various dynamic and isometric methods, including the rate of force development (RFD), which reflects the change in force relative to time; instantaneous force at specific time points; and time-specific impulse, defined as the area under a segment of the force–time curve [11]. In most strength–power sports, the key performance moments happen within very short time frames (less than 250 ms). Early force production is often further divided into early-phase (0–100 ms) and late-phase (100–250 ms) segments. The early phase is thought to primarily reflect neural factors, such as motor unit recruitment and firing frequency, whereas the late phase is determined by both neural and muscular properties, including muscle cross-sectional area and tendon stiffness [12]. If greater force can be generated during this brief period—thanks to a higher RFD—it enables increased acceleration and velocity [13].

The 0–250 ms window corresponds to typical contraction durations observed in throwing movements and other explosive actions, and its use is well established in profiling the rate of force development in strength–power athletes [11,12,14].

Track and field throwing events—javelin, hammer throw, discus, and shot put—are high-intensity disciplines requiring precise technique and rapid force production within a brief time window, typically around 250 ms or less [14,15]. Although each event has unique characteristics, all throwing disciplines place a strong emphasis on resistance training to develop power and strength. The bench press and bench row are staple exercises frequently included in the training routines of elite throwers [16].

While dynamic strength assessments are common in performance testing, isometric force assessments offer distinct advantages—such as higher reliability, lower technical demands, and reduced injury risk [17]. However, the specific relationship between early force production and maximal upper-body strength remains underexplored in elite throwers. Nonetheless, the theoretical relevance of early force production is well supported by research in similar strength–power contexts. In sports where critical actions occur within short time frames (e.g., jumping, sprinting, and Olympic weightlifting), the ability to produce high force rapidly has been associated with performance outcomes [11,12,14]. Throwers, likewise, generate substantial force within short time windows—typically between 150 and 240 milliseconds—resulting in high RFDs [11,18]. Investigating the association between early force production and maximal upper-body strength in elite throwers would therefore be valuable. More specifically, it would allow for the determination of how maximal strength (peak force) and early isometric upper-body force production at specific time points are characteristically related in successful (i.e., elite) track and field throwers of both sexes. The findings may help coaches fine-tune training strategies, ultimately enhancing athletic performance.

While the four throwing disciplines differ in technique and specific biomechanical demands, upper-body pushing and pulling actions are consistently targeted in training across events. Exercises such as the bench press and bench row are commonly used to develop upper-body strength in both male and female throwers, regardless of discipline. As such, assessing isometric push and pull force in a combined cohort may offer relevant insights into upper-body force production in this population while recognizing that the functional contributions of these actions may vary across events.

Therefore, the aim of this study was to determine the relationship between early (50, 100, 150, 200, and 250 ms) and maximal (peak force) isometric upper-body push and pull force production among elite Swedish track and field throwers.

## 2. Materials and Methods

### 2.1. Study Design, Experimental Procedures, and Test Device

This cross-sectional study involved a single testing session for each participant. A custom-built device was used to assess maximal isometric force during the bench press and supine bench row exercises, using load cells to capture force output. The purpose of this approach was to evaluate early (50, 100, 150, 200, and 250 ms) and maximal (peak force) isometric upper-body push and pull force production among elite Swedish field throwers. The custom test device has been described previously and demonstrated excellent test–retest reliability [16]. In that study, the device showed intraclass correlation coefficients ranging from 0.93 to 0.96 and coefficients of variation between 3.3 and 3.5 percent for both push and pull conditions. Briefly, the device measures isometric bench press and bench row performance from an identical test position, using separate load cells for the left and right sides while the participant lies supine on a training bench. The design relies on bi-directional load cells that measure both tension and compression forces, corresponding to push and pull forces. Bench press and bench row efforts were assessed at a neutral, midpoint position—defined as 50% of the full range of motion (ROM)—with fully extended arms representing 100% ROM.

### 2.2. Participants

A total of 30 elite Swedish track and field throwers (17 females, 13 males; aged 18–34 years) participated in this study. All competed in hammer, javelin, discus, or shot put at the national or international level (Table 1). Inclusion criteria required participants to be highly trained and familiar with the isotonic bench press and bench row exercises. Approval for this study was received from the Swedish Ethical Review Authority.

### 2.3. Procedures

**Figure 1:**
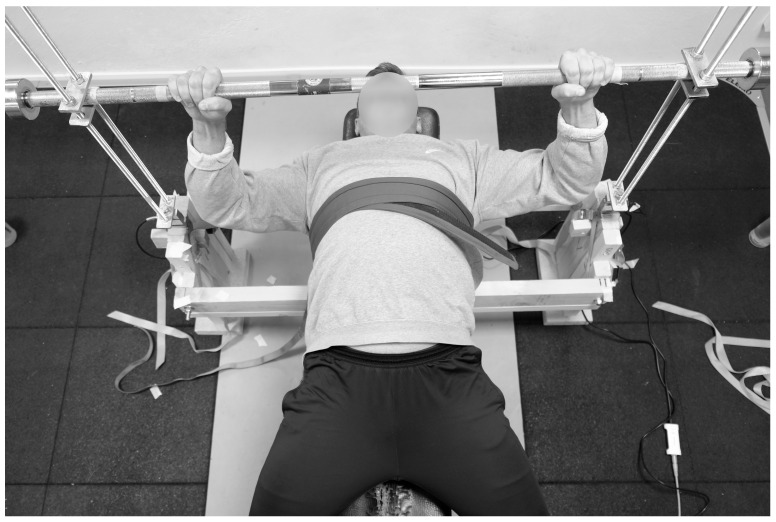
The testing setup. The participant’s test positions for the bench press (push) and supine row (pull) are depicted. For the supine bench row, the upper body was secured against the bench by a heavy-duty Velcro fixation belt. This custom-built setup has previously been described and validated for use in elite throwers [16].

Participants began with a preparatory 5 min upper-body warm-up consisting of arm and shoulder rolls and open-arm crisscross movements. They then assumed the bench press position, grasping the barbell with fully extended arms using a standardized 81 cm grip width (Figure 1). The test leader determined the vertical distance between the barbell and the chest—corresponding to the full ROM—by means of a measuring stick. Each participant then performed two isometric warm-up trials (push and pull) at approximately 50% effort at the midpoint of their ROM. Isometric force data were subsequently collected with the barbell positioned at 50% ROM for both exercises (bench press/push and supine bench row/pull).

Data from the load cells (Ergotest Technology AS, Langesund, Norway) were collected via the MuscleLab system (V10.21, Ergotest Technology AS, Langesund, Norway). Although separate load cells were used for the right and left sides, only the combined total force was used for all analyses. Force output was calculated at specific time points—50, 100, 150, 200, and 250 ms—as well as peak isometric force. The RFD (∆Force/∆Time) was also computed as the average tangential slope of the force–time curve across designated intervals: 0–50, 0–100, 0–150, 0–200, and 0–250 ms. These time intervals were selected due to their potential relevance to force generation in throwing movements [19,20]. Force onset was manually identified using the MuscleLab system (V10.21, Ergotest Technology AS, Langesund, Norway) software by a single experienced investigator, following the protocol described by Tillin et al. [21].

Participants received a verbal countdown (“3, 2, 1, push” or “pull,” depending on the test), followed by instructions to exert maximal force against the bar as quickly and forcefully as possible for 4 s. Due to the participants’ strength training background and the straightforward nature of the tests, only one to two trials were performed per condition, with two-minute rest intervals between attempts. For the supine bench row, the upper body was secured against the bench by a heavy-duty Velcro fixation belt. Test order was counterbalanced across participants, with half completing the bench press test before the supine bench row and the other half in the reverse sequence. Randomization was conducted using Microsoft Excel’s (version 2504) RAND function to ensure an even distribution.

A sports physical therapist with over 25 years of experience in strength testing and training supervised all assessments.

### 2.4. Statistical Analyses

Statistical analyses were conducted using IBM SPSS Statistics (version 30, IBM, Armonk, NY, USA). The normality of the data was confirmed via the Shapiro–Wilk test *(p* > 0.05), justifying the use of parametric tests. The results are reported as means with standard deviations (SDs). Pearson product-moment correlation coefficients were used to examine the associations between early (50, 100, 150, 200, and 250 ms) and maximal (peak force) isometric upper-body push and pull force production among elite track and field throwers of both sexes. The strength of the correlations was interpreted using the following criteria: *r* = 0.00–0.10, insignificant; *r* = 0.10–0.39, weak; *r* = 0.40–0.69, moderate; *r* = 0.70–0.89, strong; and *r* = 0.90–1.00, very strong [22].

Statistical significance was defined as *p* < 0.05.

## 3. Results

Descriptive statistics for early and maximal isometric push and pull force variables, including force output at defined time intervals expressed as a percentage of peak force, are shown in Table 2. Push-to-pull strength ratios at 50% of the full ROM were 1.05 for female throwers and 1.12 for male throwers.

The results indicated a stepwise increase in the correlation between early and maximal force production for both pushing (bench press) and pulling (bench row) actions. As shown in Table 3, correlations with peak force ranged from non-significant (*p* > 0.05, *r* = 0.07–0.55) at 50 ms to significant *(p* < 0.05, *r* = 0.64–0.92) at 250 ms, for both tests and across female and male throwers.

**Figure 2:**
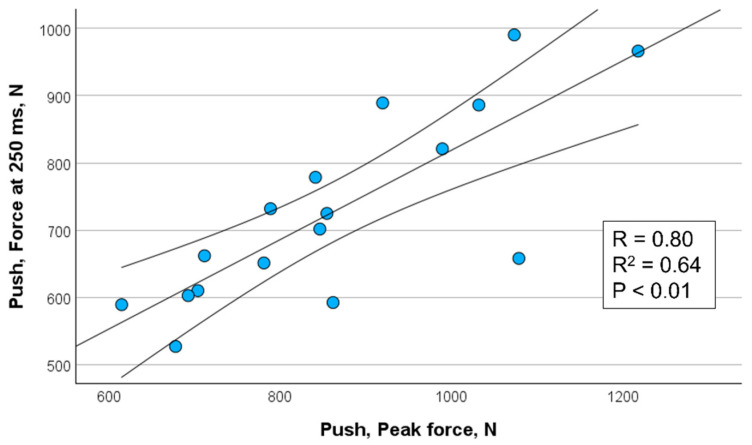
Correlation between peak force and force measured at 250 ms for push (isometric bench press) in female throwers (*n* = 17) including 95% confidence interval.

**Figure 3:**
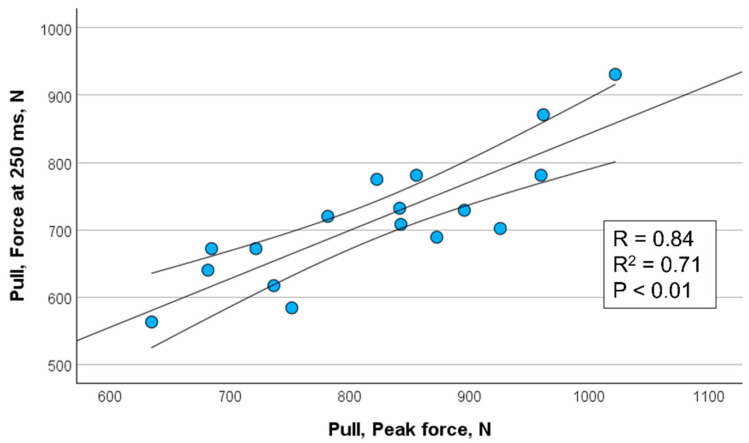
Correlation between peak force and force measured at 250 ms for pull (isometric bench row) in female throwers (*n* = 17) including 95% confidence interval.

**Figure 4:**
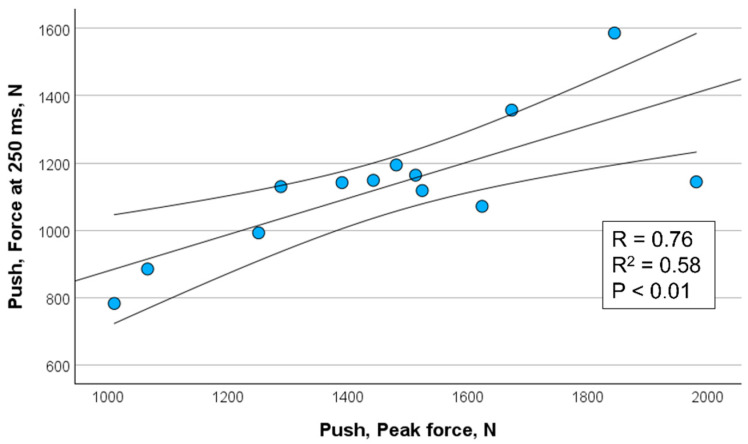
Correlation between peak force and force measured at 250 ms for push (isometric bench press) in male throwers (*n* = 13) including 95% confidence interval.

**Figure 5:**
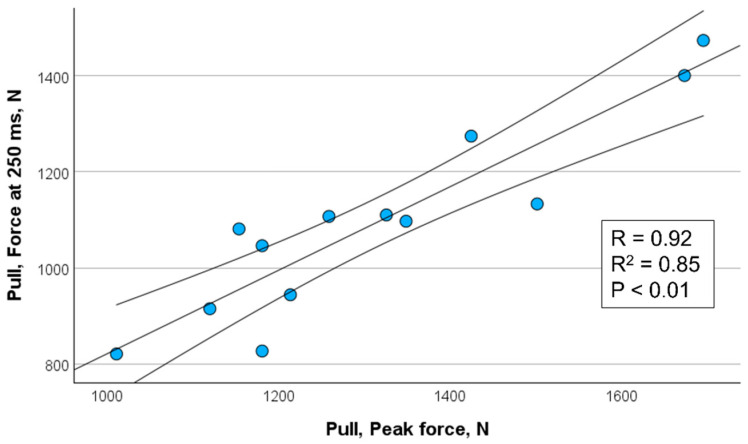
Correlation between peak force and force measured at 250 ms for pull (isometric bench row) in male throwers (*n* = 13) including 95% confidence interval.

Figure 2, Figure 3, Figure 4 and Figure 5 show the significant relationships between measures of peak force and force at 250 ms for push (isometric bench press) and pull (isometric bench row) across all throwers.

## 4. Discussion

The findings of this study indicate a clear and progressively stronger relationship between early and maximal isometric force production in elite throwers, particularly from 150 ms onward. This pattern was evident across both push and pull movements and in both sexes. The results reinforce the notion that maximal strength serves not only as a performance determinant on its own but also as a foundational contributor to an athlete’s capacity to rapidly generate force—a key component in explosive tasks such as throwing.

The progressively stronger associations observed across increasing time intervals reflect the physiological distinction between early- and late-phase force production. Early-phase force (0–100 ms) is primarily influenced by neural factors, including motor unit firing frequency and recruitment, while force output beyond 100 ms increasingly reflects musculotendinous contributions [12,23]. This framework helps explain why statistically significant correlations with peak force first emerged at or beyond 150 ms in the current study. It also aligns with prior work suggesting that the majority of force application in explosive athletic actions occurs within a critical window of 150–250 ms [11,14]. From a neuromuscular perspective, this underscores the importance of maximal strength for generating force rapidly in time-constrained efforts—particularly relevant in throwing events where implement release occurs within such brief time frames.

Because the 250 ms time point showed the strongest association with peak force among the time intervals assessed, it was selected for visualization in Figure 2, Figure 3, Figure 4 and Figure 5. This decision also aligns with the previous literature emphasizing the importance of late-phase force expression (≥150 ms) in relation to strength and power performance (e.g., [11,12]). The selective presentation of this time point enhanced visual clarity and avoided redundancy across closely spaced late-phase intervals.

The custom-built push and pull testing device, previously described in detail, has been shown to exhibit excellent reliability in elite athletes [16]. Importantly, these outcomes were obtained without any prior familiarization sessions for the participants. This suggests that the measurement of isometric push and pull force at mid-range joint angles (i.e., approximately 50% of the full range of motion) is both feasible and consistent in highly trained populations. Mid-range of motion isometric testing offers a mechanically neutral position that balances joint torque and minimizes joint angle variability across individuals, thereby improving test standardization and comparability. The strong agreement with previous reports on the reliability of other isometric tests, such as the isometric mid-thigh pull [17], isometric knee extension [24], and knee flexion tests [25], reinforces the methodological soundness of this approach. Static testing conditions reduce the impact of technical execution variability, allowing the athlete to concentrate their effort solely on maximal voluntary contraction, which likely contributes to measurement reliability.

Although the isometric bench press and supine bench row are not biomechanically specific to the rotational and dynamic nature of throwing, they remain highly relevant from a strength development perspective. These exercises are commonly used in training programs for throwers to target upper-body pushing and pulling strength, which underpins force generation in more complex sport-specific actions [16,26]. Moreover, the isometric testing of these movements offers a controlled, low-fatigue, and reliable method for assessing force characteristics, particularly in high-level athletes where testing time and injury risk must be minimized [17,27]. While the results may not translate directly to throwing performance, they contribute to profiling general neuromuscular capabilities that support overall athletic development.

An interesting observation from the descriptive data (Table 2) is the remarkable similarity between push and pull force values across both sexes throughout the force–time curve, including peak force. Despite biomechanical differences between horizontal pushing and pulling actions, absolute force output and the rate of force development were closely aligned. This suggests a relatively balanced development of upper-body pushing and pulling capacities among these elite throwers at the group level, with push-to-pull strength ratios at 50% of the full ROM of 1.05 for female throwers and 1.12 for male throwers. In line with this, Augustsson et al. [16] also reported consistent push–pull strength ratios in top-tier field throwers, reinforcing that well-rounded upper-body training may be a feature of this athletic population. Moreover, balanced agonist–antagonist strength in overhead and rotational athletes has shown associations with both enhanced performance and reduced injury risk [28]. The similarity across sexes in relative performance patterns may reflect common neuromuscular adaptations or shared training strategies—possibly representing a deliberate approach to optimizing both performance and resilience. In addition to its relevance for performance, balanced upper-body force production—as observed in this cohort—may also contribute to shoulder health and reduce injury risk, with potential positive implications for long-term musculoskeletal health and career longevity, particularly in high-load overhead athletes.

While Table 2 presents comprehensive descriptive statistics for early and maximal isometric push and pull force, no formal hypothesis testing was conducted for these variables. This decision reflects a deliberate and conservative analytical approach, aimed at avoiding the inflated risk of Type I error that arises from conducting multiple significance tests across numerous variables—a phenomenon often referred to as “mass significance” or the multiple comparisons problem [29]. Instead, a descriptive presentation offers a transparent overview of group-level patterns without inflating statistical significance. This aligns with contemporary recommendations in the literature advocating for a shift away from indiscriminate *p*-value reporting toward more informative and context-sensitive data interpretation [30,31]. Presenting the data descriptively provides valuable insight while maintaining statistical integrity, particularly in exploratory or multifactorial performance research settings.

In addition to absolute values, the time-specific force outputs expressed as percentages of peak force (Table 2) may serve as informative and easily interpretable reference points for both coaches and athletes. These relative measures indicate how much of an athlete’s maximal force can be generated within specific time frames (e.g., 100 or 150 ms), providing a clear and intuitive snapshot of rapid force production capacity. While the RFD remains a commonly reported metric for assessing explosive strength [12], it is often abstract and less accessible to practitioners due to its mathematical complexity and unit expression (e.g., N·s^−1^). In contrast, percent-of-peak force values offer a more straightforward interpretation by capturing how rapidly an athlete can generate force relative to their maximum. This form of normalization is conceptually similar to the Dynamic Strength Index (DSI), which compares dynamic force production to maximal isometric strength to profile neuromuscular capabilities and guide training interventions [32]. As emphasized by Suchomel et al. [33], indices such as the DSI help contextualize performance variables and inform individualized programming—suggesting that percent-of-peak force values may serve a similarly useful function within isometric testing protocols.

From a practical standpoint, the findings support the integration of both maximal and early isometric force assessments into regular athlete monitoring. This aligns with the conclusions of Lum and Aziz [26], who found that isometric force–time characteristics—such as peak force and the RFD—are significantly correlated with dynamic performance outcomes in both upper- and lower-body movements. These tests may serve as valuable tools not only for strength profiling but also for guiding individualized training strategies. For instance, athletes with relatively low force at 100–150 ms relative to their peak may benefit from targeted interventions aimed at enhancing the RFD—such as high-velocity strength training, plyometrics, or resisted throws—although this warrants further investigation. This is consistent with the findings of Garrett and Lutton [34], who reported that youth athletes with high relative RFD ratios performed better in dynamic sports assessments, suggesting that the RFD is a key determinant of athletic performance.

Conversely, athletes with high early force outputs but relatively lower maximal strength might prioritize heavier, lower-velocity strength work to raise their ceiling. This approach is supported by the idea that different force–time profiles require tailored interventions to optimize performance outcomes, as highlighted in the systematic review by Lum et al. [27].

Additionally, the strong correlation observed at later time points (200–250 ms) highlights the potential utility of isometric testing for detecting changes over time, such as adaptations following a strength–power training cycle or periods of reduced load. This is echoed in research showing that isometric mid-thigh pull and squat tests are sensitive to neuromuscular adaptations and can serve as reliable indicators of training status. For example, Giles et al. [35] demonstrated that isometric force–time characteristics, particularly at later time intervals, are strongly associated with dynamic performance and are responsive to training-induced changes, reinforcing their value in longitudinal athlete monitoring.

This study does, however, have limitations. The generalizability of the findings may be restricted, as the sample consisted exclusively of elite male and female track and field throwers. Additionally, the relatively small sample size limited the ability to make robust comparisons between the different throwing disciplines (discus, hammer, javelin, and shot put). Nevertheless, the athlete group constitutes a considerable share of Sweden’s high-performance throwing population.

### 4.1. Practical Applications

Coaches and strength practitioners can use time-specific isometric force measures to evaluate and monitor both early force capacity and peak output in elite throwers. Because strong correlations with peak force emerge from 150 ms onward, interventions aiming to improve maximal strength may also enhance late-phase explosive force. For athletes with early-phase deficits, incorporating high-intensity ballistic exercises (e.g., medicine ball throws, bench press throws, or speed-strength loading) may be beneficial. The regular use of isometric mid-range testing can help guide training priorities and detect adaptation plateaus, offering a low-fatigue alternative to dynamic assessments.

### 4.2. Future Directions

Future studies should explore how time-specific isometric force variables correlate with actual throwing performance and competition outcomes. Longitudinal studies examining how changes in isometric profiles relate to strength–power periodization would further clarify the utility of this method in applied settings. Additionally, expanding the sample to include different age groups and competitive levels could improve the generalizability of the findings.

Further research should also investigate whether isometric force characteristics—particularly balanced push–pull profiles—are associated with reduced injury rates, improved shoulder function, and long-term musculoskeletal health among throwing athletes.

## 5. Conclusions

This study highlights the progressively stronger association between early and maximal isometric upper-body force production in elite throwers, particularly beyond 150 ms. These findings support the inclusion of time-specific isometric testing as a complementary tool for assessing force development capacity. While the current study does not evaluate training outcomes, the observed relationships may help inform strength training decisions or guide individualized programming in practice.

## Figures and Tables

**Table 1:** Participants’ characteristics (*n* = 30).

Characteristics	Female (*n* = 17)	Male (*n* = 13)
Age (years)	24 ± 4	23 ± 5
Height (cm)	177 ± 6	188 ± 6
Body mass (kg)	78 ± 10	104 ± 13
Practice (hours/week)	14 ± 3	15 ± 4
Right-hand dominant	17	12
Discus (PB range, m)	5 (51.0–59.5)	4 (51.5–59.0)
Hammer (PB range, m)	5 (63.0–72.5)	2 (64.5–67.0)
Shot put (PB range, m)	2 (15.0–15.5)	2 (17.5–18.5)
Javelin (PB range, m)	5 (51.5–55.5)	5 (70.0–75.0)

PB = personal best. To ensure participant confidentiality, individual personal bests were rounded to the nearest 0.5 m before presentation.

**Table 2:** Descriptive statistics presented as mean values (SD), including force output at defined time intervals expressed as a percentage of peak force, for early and maximal isometric push and pull force in the bench press and supine bench row assessments conducted on elite female (*n* = 17) and male (*n* = 13) throwers.

Force Variable	Females, Bench Press	Females, Bench Row	Males, Bench Press	Males, Bench Row
Peak force, N	864 (168)	823 (111)	1468 (279)	1315 (210)
Force @ 50 ms, N	339 (148)	299 (94)	541 (215)	526 (149)
Force @ 100 ms, N	521 (169)	509 (92)	829 (279)	840 (168)
Force @ 150 ms, N	626 (160)	623 (80)	965 (251)	989 (173)
Force @ 200 ms, N	690 (146)	679 (87)	1053 (200)	1058 (181)
Force @ 250 ms, N	728 (140)	716 (95)	1132 (198)	1094 (199)
Force @ 50 ms, %	39	36	37	40
Force @ 100 ms, %	60	62	56	64
Force @ 150 ms, %	72	76	66	75
Force @ 200 ms, %	80	83	72	80
Force @ 250 ms, %	84	87	77	83
RFD, 0–50 ms, N/s	6799 (3036)	6062 (2149)	10,587 (4680)	10,633 (2878)
RFD, 0–100 ms, N/s	5252 (1755)	5204 (1096)	8396 (3289)	8371 (1480)
RFD, 0–150 ms, N/s	4028 (1005)	4220 (654)	6215 (1906)	6422 (1098)
RFD, 0–200 ms, N/s	3194 (636)	3342 (565)	4797 (1117)	4932 (960)
RFD, 0–250 ms, N/s	2578 (469)	2683 (507)	3857 (823)	3847 (894)

RFD = rate of force development.

**Table 3:** The relationships between peak force and force values at 50, 100, 150, 200, and 250 ms were examined using Pearson correlation coefficients (*r*) and coefficients of determination (*r*^2^) for both push (isometric bench press) and pull (isometric bench row) strength in elite female (*n* = 17) and male (*n* = 13) throwers.

	Females, Push Peak Force, N	Males, Push Peak Force, N	Females, Pull Peak Force, N	Males, Pull Peak Force, N
Force @ 50 ms, N	*r* = 0.29 *r*^2^ = 0.08	*r* = 0.27 *r*^2^ = 0.07	*r* = 0.07 *r*^2^ = 0.01	*r* = 0.55 *r*^2^ = 0.30
Force @ 100 ms, N	*r* = 0.50 * *r*^2^ = 0.25	*r* = 0.43 *r*^2^ = 0.19	*r* = 0.33 *r*^2^ = 0.11	*r* = 0.78 ** *r*^2^ = 0.61
Force @ 150 ms, N	*r* = 0.61 ** *r*^2^ = 0.37	*r* = 0.54 *r*^2^ = 0.29	*r* = 0.67 ** *r*^2^ = 0.45	*r* = 0.89 ** *r*^2^ = 0.79
Force @ 200 ms, N	*r* = 0.71 ** *r*^2^ = 0.50	*r* = 0.65 * *r*^2^ = 0.42	*r* = 0.78 ** *r*^2^ = 0.61	*r* = 0.92 ** *r*^2^ = 0.85
Force @ 250 ms, N	*r* = 0.80 ** *r*^2^ = 0.64	*r* = 0.76 ** *r*^2^ = 0.58	*r* = 0.84 ** *r*^2^ = 0.71	*r* = 0.92 ** *r*^2^ = 0.85

* The correlation is statistically significant at the 0.05 level. ** The correlation is statistically significant at the 0.01 level.

## Data Availability

The data supporting the findings of this study are available from the corresponding author upon request.

## References

[1] American College of Sports Medicine (2009). American College of Sports Medicine position stand. Progression models in resistance training for healthy adults. Med. Sci. Sports Exerc..

[2] Stone M.H., Hornsby W.G., Suarez D.G., Duca M., Pierce K.C. (2022). Training Specificity for Athletes: Emphasis on Strength-Power Training: A Narrative Review. J. Funct. Morphol. Kinesiol..

[3] Redman K.J., Kelly V.G., Beckman E.M. (2021). Seasonal Changes in Strength and Power in Elite Rugby League: A Systematic Review and Meta-Analysis. J. Sports Sci. Med..

[4] Beckham G., Mizuguchi S., Carter C., Sato K., Ramsey M., Lamont H., Hornsby G., Haff G., Stone M. (2013). Relationships of isometric mid-thigh pull variables to weightlifting performance. J. Sports Med. Phys. Fit..

[5] Kirkpatrick J., Comfort P. (2013). Strength, power, and speed qualities in English junior elite rugby league players. J. Strength Cond. Res..

[6] Seitz L.B., Reyes A., Tran T.T., Saez de Villarreal E., Haff G.G. (2014). Increases in lower-body strength transfer positively to sprint performance: A systematic review with meta-analysis. Sports Med..

[7] Styles W.J., Matthews M.J., Comfort P. (2016). Effects of Strength Training on Squat and Sprint Performance in Soccer Players. J. Strength Cond. Res..

[8] Aagaard P., Simonsen E.B., Andersen J.L., Magnusson P., Dyhre-Poulsen P. (2002). Increased rate of force development and neural drive of human skeletal muscle following resistance training. J. Appl. Physiol..

[9] Andersen L.L., Aagaard P. (2006). Influence of maximal muscle strength and intrinsic muscle contractile properties on contractile rate of force development. Eur. J. Appl. Physiol..

[10] Suchomel T.J., Nimphius S., Stone M.H. (2016). The Importance of Muscular Strength in Athletic Performance. Sports Med..

[11] Turner A.N., Comfort P., McMahon J., Bishop C., Chavda S., Read P., Mundy P., Lake J. (2020). Developing Powerful Athletes, Part 1: Mechanical Underpinnings. Strength Cond. J..

[12] Maffiuletti N.A., Aagaard P., Blazevich A.J., Folland J., Tillin N., Duchateau J. (2016). Rate of force development: Physiological and methodological considerations. Eur. J. Appl. Physiol..

[13] Stone M.H., Pierce K.C., Sands W.A., Stone M.E. (2006). Weightlifting: A Brief Overview. Strength Cond. J..

[14] Zaras N.D., Stasinaki A.N., Methenitis S.K., Krase A.A., Karampatsos G.P., Georgiadis G.V., Spengos K.M., Terzis G.D. (2016). Rate of Force Development, Muscle Architecture, and Performance in Young Competitive Track and Field Throwers. J. Strength Cond. Res..

[15] Stone M.H., Sanborn K., O’Bryant H.S., Hartman M., Stone M.E., Proulx C., Ward B., Hruby J. (2003). Maximum strength-power-performance relationships in collegiate throwers. J. Strength Cond. Res..

[16] Augustsson J., Gunhamn T., Andersson H. (2024). An Assessment of the Ratio between Upper Body Push and Pull Strength in Female and Male Elite Swedish Track and Field Throwers. Sports.

[17] Grgic J., Scapec B., Mikulic P., Pedisic Z. (2022). Test-retest reliability of isometric mid-thigh pull maximum strength assessment: A systematic review. Biol. Sport.

[18] Zaras N., Stasinaki A.N., Terzis G. (2021). Biological Determinants of Track and Field Throwing Performance. J. Funct. Morphol. Kinesiol..

[19] Bartlett R.M., Best R.J. (1988). The biomechanics of javelin throwing: A review. J. Sports Sci..

[20] Zatsiorsky V.M., Lanka G.E., Shalmanov A.A. (1981). Biomechanical analysis of shot putting technique. Exerc. Sport. Sci. Rev..

[21] Tillin N.A., Jimenez-Reyes P., Pain M.T., Folland J.P. (2010). Neuromuscular performance of explosive power athletes versus untrained individuals. Med. Sci. Sports Exerc..

[22] Schober P., Boer C., Schwarte L.A. (2018). Correlation Coefficients: Appropriate Use and Interpretation. Anesth. Analg..

[23] Rodríguez-Rosell D., Pareja-Blanco F., Aagaard P., González-Badillo J.J. (2018). Physiological and methodological aspects of rate of force development assessment in human skeletal muscle. Clin. Physiol. Funct. Imaging.

[24] Venegas-Carro M., Kramer A., Moreno-Villanueva M., Gruber M. (2022). Test-Retest Reliability and Sensitivity of Common Strength and Power Tests over a Period of 9 Weeks. Sports.

[25] Ogborn D.I., Bellemare A., Bruinooge B., Brown H., McRae S., Leiter J. (2021). Comparison of Common Methodologies for the Determination of Knee Flexor Muscle Strength. Int. J. Sports Phys. Ther..

[26] Lum D., Aziz L. (2020). Validity and Reliability of the Isometric Prone Bench Pull Test. Int. J. Sports Med..

[27] Lum D., Haff G.G., Barbosa T.M. (2020). The Relationship between Isometric Force-Time Characteristics and Dynamic Performance: A Systematic Review. Sports.

[28] Ellenbecker T.S., Cools A. (2010). Rehabilitation of shoulder impingement syndrome and rotator cuff injuries: An evidence-based review. Br. J. Sports Med..

[29] Perneger T.V. (1998). What’s wrong with Bonferroni adjustments. BMJ.

[30] Wasserstein R.L., Lazar N.A. (2016). The ASA Statement on p-Values: Context, Process, and Purpose. Am. Stat..

[31] Amrhein V., Greenland S., McShane B. (2019). Scientists rise up against statistical significance. Nature.

[32] Thomas C., Jones P.A., Comfort P. (2015). Reliability of the Dynamic Strength Index in college athletes. Int. J. Sports Physiol. Perform..

[33] Suchomel T.J., Sole C.J., Bellon C.R., Stone M.H. (2020). Dynamic Strength Index: Relationships with Common Performance Variables and Contextualization of Training Recommendations. J. Hum. Kinet..

[34] Garrett C.G., Lutton G. (2024). The Relationship Between Isometric Rate of Force Development and Isometric Maximum Strength Ratio to Dynamic Performance in Youth Athletes. Res. Investig. Sports Med..

[35] Giles G., Lutton G., Martin J. (2022). Scoping Review of the Isometric Mid-Thigh Pull Performance Relationship to Dynamic Sport Performance Assessments. J. Funct. Morphol. Kinesiol..

